# Disclosure of Sibling Sexual Abuse by Hospitalized Adolescent Girls: Three Case Reports

**DOI:** 10.3389/fpsyt.2021.792012

**Published:** 2022-01-25

**Authors:** Emilie Carretier, Jonathan Lachal, Nina Franzoni, Selim Benjamin Guessoum, Marie Rose Moro

**Affiliations:** ^1^Assistance Publique des Hôpitaux de Paris, Hôpital Cochin, Maison de Solenn, Paris, France; ^2^Université de Paris, Département Laboratoire de Psychologie Clinique Psychopathologie Psychanalyse, Boulogne-Billancourt, France; ^3^University Paris-Saclay, UVSQ, INSERM, CESP, Team ≪ DevPsy ≫, Villejuif, France; ^4^Département de Psychiatrie de l'Enfant et de l'Adolescent, Centre Hospitalo-Universitaire de Clermont-Ferrand, Clermont-Ferrand, France; ^5^Université Clermont Auvergne, Département de Médecine, Clermont-Ferrand, France

**Keywords:** sibling sexual abuse, sibling incest, child sexual abuse, child protection, disclosure, adolescent psychiatry, case report

## Abstract

Although sibling sexual abuse (SSA) may be the most common type of intrafamilial sexual abuse, it has not been widely studied. The lack of studies makes it very difficult for clinicians to create a comprehensive framework about this complex phenomenon, particularly in comparison with other forms of intrafamilial sexual abuse, such as father-daughter incest. SSA is still underrecognized and underdisclosed but it has the potential to be every bit as harmful as sexual abuse by a parent. The topic rarely finds its way into the more general psychiatry or social work literature. It is imperative to increase healthcare practitioners' awareness of this complex subject to improve their ability to listen to, detect, and manage the disclosures of SSA in adolescent populations. This paper presents vignettes of three 13-to-15-year-old adolescent girls who disclosed SSA during inpatient hospitalization in an adolescent psychiatric and medicine department. These cases illustrate the complexity of SSA, which has been associated with a wide spectrum of both mental and physical symptoms. Adolescent victims of SSA experience serious distress, with various and numerous psychiatric manifestations, including but not limited to depression and suicide attempts, addictive behaviors, post-traumatic stress symptoms, and eating disorders. Physical symptoms should also alert practitioners: adolescent survivors are more likely to be affected by somatic complications such as sexually transmitted diseases, chronic pain, urogenital symptoms, and nutritional disorders. We offer some recommendations to improve the detection and support of distressed adolescents disclosing SSA. Listening to them and offering a protective multidisciplinary response can limit the lasting damage and contribute to the repair process.

## Introduction

Sibling sexual abuse (SSA) is defined as sexual behavior between two or more siblings that is age-inappropriate and not motivated by developmental or appropriate curiosity (1–3). Caffaro defines it as sexual acts initiated by one sibling (brother or sister) toward another (brother or sister) without the other's consent, by use of force or coercion, or where there is a power differential between the children. It may involve children of similar or different ages; aggression, coercion, or force; harm or the potential for harm; may occur frequently or infrequently; and may include minor or advanced sexual behaviors (4). Sexual behavior includes both non-contact behaviors, such as watching pornography, and physical contact behaviors, ranging from fondling to forcible penetration (2, 5). Although SSA may be the most common type of intrafamilial sexual abuse (6–8), the lack of studies makes it very difficult for clinicians to create a comprehensive framework about this complex phenomenon (9–11), particularly in comparison with other forms of intrafamilial sexual abuse, such as father-daughter incest (12, 13). Yates (14) notes that most SSA-related papers are published in journals that are specifically concerned with child abuse or journals concerned with families and family therapy. The topic rarely finds its way into the more general psychiatry or social work literature. It is therefore essential to increase healthcare practitioners' awareness of this complex subject to improve their ability to listen to, detect, and manage SSA disclosure in adolescent populations. Any statistics relating to SSA prevalence are likely to be underestimated, because it is rarely disclosed and is reported less often than sexual abuse by adults (8, 14). Survivors may not disclose SSA due to fears of punishment, blame, or not being believed, or because they are afraid of the sibling, do not identify the SSA as an aggression, want to avoid trouble for their sibling or upsetting their parents, or just want no one to know about it (15, 16). Moreover SSA is the form of sexual abuse least often reported to social workers or law enforcement authorities, perhaps because parents and guardians believe it is harmless, consensual, and even a normal part of childhood sexual explorations (11, 12, 17, 18). However, it has traumatic effects with psychological, emotional, and physical consequences that may be both devastating and long-lasting, at least as much as the abuse of a child by a parent (5, 8, 19). Childhood sexual abuse is associated with psychiatric disorders, severe functional impairments, and substantially lower quality of life during adulthood (1, 8, 13, 20).

The aim of this report is to increase awareness of SSA among healthcare practitioners by presenting three illustrative cases of adolescent girls who disclosed long-term SSA during inpatient hospitalization in an adolescent psychiatric and medicine department. In addition, this report aims to summarize the available scientific evidence on the importance of disclosure and care for adolescent survivors of SSA and to discuss how to improve its detection as well as support for distressed adolescents disclosing it. Informed consent was obtained from each of the patients and their parents.

### Case 1

Emma, aged 13 years, was hospitalized for an evaluation at the request of her psychiatrist. She had been completely out of school for the past 18 months, socially withdrawn, and spent most of her time playing video games in her bedroom. Emma was living with her mother and her 15-year-old brother, who has a psychiatric condition with psychotic symptoms. Her parents divorced when she was 6 years-old and she has stopped seeing her father two years ago. When we met Emma, she was very inhibited, had a distant, absent look, and bradyphemia. Her body showed both old and recent scars from cutting on her arms, forearms, legs, and thighs. She explained that she needed to cut herself when she was very distressed, but she was unable to say what triggered this distress. She spent her days in her room at home, saying that she felt safe there, compared with anywhere else. During her hospitalization, when she was moved from a single room to a double room with another girl, a resurgence of cutting, anxiety, and unkempt appearance occurred. When we asked Emma about her feelings of insecurity, she spoke spontaneously about the period when she had been seeing her father and how she felt he demeaned or humiliated her. When we asked her if she had experienced other hurtful events, she shook her head, staring blankly. A few days later she disclosed to a nurse that her older brother had repeatedly raped her. The most recent episode had taken place about 2 years earlier, on a vacation with their father. Emma reported that since this abuse began, she had had daily traumatic flashbacks. Her disclosure helped us understand her dissociative symptoms, caused by a complex post-traumatic stress disorder. After our report to Child Protective Services, Emma was interviewed by the Police Division for child protection and later by a Family Court judge. She was represented by a lawyer throughout the legal proceeding. Because the judge did not want to separate either sibling from the mother, Emma had to return home. On discharge, she continued pharmacologic treatment (antidepressant sertraline 75 mg per day and risperidone 1 mg per day) as well as psychotherapy. Her mother organized alternating custody with the aunt and grandparents to separate Emma from her brother. Her dissociative symptoms decreased significantly, and she has succeeded in returning to school.

### Case 2

Rachel, aged 15 years, was admitted to our department for a multidisciplinary evaluation of chronic pain, depressive symptoms, and refusal to attend school. Work-ups during hospitalization did not identify any organic explanations for the chronic arthritic pain. The psychiatric evaluation concluded by a diagnosis of general anxiety disorder and binge eating disorder. During a psychiatric interview, she explained that the full symptoms had begun about six months earlier and had worsened during the first COVID-19 lockdown (March-May 2020). She described difficulty coping with family confinement because of significant interpersonal conflict. Rachel's anxiety increased after this session, with several panic attacks and the expression of suicidal thoughts. She finally wrote a letter to the psychiatrist explaining that she had experienced sexual activity with her older brother, over a two-year period when she was aged 12–14 years and her brother 14–16. She wrote ≪ *Sometimes he forced me to do things that I didn't really want to do. He touched my private parts. Several times he “entered” me. I must admit I didn't say anything because I thought that this “relationship” was ‘'normale”, but sometimes I didn't want to do certain things or the act itself and he knew it but he said to me that I would know how to do it later when I wanted to* ≫. In her letter, she expressed relief about freeing herself of this secret kept for several years, but also fear of rejection by her parents: ≪ *It's good for me to talk about it and to let it out because I've been holding it in for all these years. What would destroy me even more is if there were repercussions toward my parents, my brother, my family.... I would rather die than live if everyone in my own family hates me*. ≫ The hospitalization was extended to work with Rachel and her parents, before a possible return home. Upon discharge, psychiatric and psychotherapeutic follow-up began. Rachel returned for short hospitalizations for reassessment and therapeutic support. Antidepressant treatment (sertraline 50 mg per day) was later initiated because of the persistent anxiety disorder with post-traumatic symptoms (reminiscences and nightmares). She finally entered a treatment facility that included schooling to enable her to return to school in an appropriate environment, to receive medical support, and to allow family work while keeping her away from her brother.

### Case 3

Kelly was hospitalized at the age of 14 years after repeated suicide attempts with drugs over a 6-month period. She had begun seeing a psychiatrist a year earlier, due to depressive symptoms with suicidal ideation, cutting, insomnia, and the emergence of bulimia nervosa symptoms. She attempted suicide by drug poisoning twice within a short interval. During her hospitalization in the pediatric emergency department after one of them, she suggested to several professionals that these suicide attempts were related to home conflicts during visits of her older brother. He was no longer living with their parents but came to visit regularly. She mentioned flashbacks of his exhibitionist and violent behavior. The consultation-liaison psychiatrist recommended hospitalization in our child psychiatry department to continue the evaluation away from the family environment. On admission, Kelly presented depressive mood, psychomotor slowing, self-induced vomiting, and suicidal ideation. She expressed no regret about her previous suicide attempts, and on arrival mentioned fears about discharge and a possible return home. She explained that prior to hospitalization, she had moved in with an older sister to avoid her older brother, who frequently visited the parental home. After a few days of hospitalization, away from her family, she disclosed verbal, physical, and sexual violence from her brother. We reported the situation to Child Protection Services after informing the parents. A medicolegal assessment was ordered after the report, and a gynecologic examination observed traces of previous trauma confirming the disclosures. SSA disclosure and management, and antidepressant treatment (fluoxetine 20 mg per day) led to remission of her symptoms. Kelly returned to her home, with a guarantee from her mother that the older brother would no longer come back. A few months later, she was moved to a foster family due to persistent insecurity at home and a lack of parental support.

## Discussion

These three case reports show that, when an adolescent feels safe enough, for example, during an inpatient hospitalization, with psychiatrists trained in the management of sexual abuse, both SSA disclosure and appropriate management are possible. These reports suggest that when such disclosure and management occur, they can contribute to psychiatric remission. Adolescent survivors of SSA experience serious distress, with various and numerous psychiatric or physical manifestations. Many researchers have highlighted the severe effects of SSA (1, 8, 19, 21, 22), also evident in our case reports.

### The Impact of Sibling Sexual Abuse

The social myth that sexual acts between siblings are mutual, harmless, and innocent, resulting from natural age-appropriate curiosity, has persisted for a long time (11). The fact that the age gap between the survivor and perpetrator is relatively small might contribute to this myth (17, 23). Nonetheless, in the early 2000s, several comparative studies showed that brother-sister sexual abuse is at least as harmful as father-daughter abuse or other adult-child abuse (19, 21, 24, 25). Hardy argues that SSA has the same psychological and emotional impact on girls as sexual abuse by a father (16).

SSA is often associated with serious psychiatric disorders (26), which should serve as signals of alert for families and professionals. Disorders with a higher prevalence among survivors of SSA include: (a) depression and suicide attempts (20, 21, 27–29); (b) addictive behaviors (19, 21, 28); (c) post-traumatic stress symptoms such as dissociation, flashbacks, nightmares, and intrusive thoughts (15, 19, 23, 27, 30); (d) emotional and behavioral disorders (24); (e) low self-esteem and ongoing feelings of guilt and shame (9, 20, 23, 27); and (f) eating disorders (bulimia nervosa > anorexia nervosa) (21, 27, 28). Physical symptoms should also alert practitioners. Adolescent victims are more likely to be affected by somatic complications such as: (a) pregnancy, or sexually transmitted diseases (31–33); (b) chronic pain (headaches, abdominal pain, and fibromyalgia); (c) urogenital symptoms (enuresis or secondary encopresis or both); and (d) nutritional disorders (obesity or diabetes or both) (34). Our case reports illustrate the spectrum of symptoms among adolescent survivors of SSA. Each reason for inpatient admission was unusual and serious: repeated suicide attempts, eating disorders, cutting, school refusal, and chronic pain syndrome. The abuse began several years before the first visible symptoms appeared. Yates explains that while sibling sexual abuse is immediately harmful, its effects may not be immediately evident to the victim or the observer (35). Finkelhor and Berliner (36) report that up to 40% of children sexually abused by adults do not present immediate symptoms, although many of them develop symptoms and more symptoms as time passes (29).

Adolescence is a period during which health professionals must be particularly attentive to disclosures of sexual violence. The transition from childhood to adulthood may reactivate old corporal trauma at the onset of puberty—a process reported to be frightening, especially to some vulnerable adolescents (37).

### The Need to Train Professionals in SSA Disclosure and Management

Our case reports also highlight the need to treat and to protect adolescent survivors. This protection requires a report to child welfare authorities together with medical and psychiatric treatment. Practitioners' difficulty in identifying SSA probably affects reporting rates (11). SSA is characterized by high levels of underreporting, even compared with other types of sexual assault (8, 28). It is estimated that only 20% of all SSA cases are reported and finally treated by health professionals (38). Survivors receive less therapeutic attention than those of other forms of intrafamilial sexual abuse (39). Most studies note that professionals fail to consider sibling sexual behavior as potentially abusive or to respond appropriately when it is disclosed (14, 25, 40, 41). The gap between how the phenomenon is perceived, even by professionals, and its actual harmful consequences creates a difficult situation for both survivors and their families, who frequently receive neither appropriate recognition of their pain nor an appropriate professional response (40). The three case reports illustrate a common theme: adolescents sibling sexual abuse survivors postdisclosure, unlike their adult counterparts, are more often likely to remain in the same home, school, and community as their offender. This important distinction between SSA and adult caregiver-child sexual abuse has been found for some time in the SSA literature (29, 42). A key decision that needs to be made is whether the siblings can continue to live together, at least until further investigation and assessment are undertaken. Separation should always be considered where there are concerns about immediate physical safety, or where the continued presence of the child who has harmed causes significant distress (43).

Disclosure, under appropriate circumstances, has important positive benefits such as stopping the sexual abuse, beginning a repair process by treating the trauma, and helping the entire family. It can also prevent the emergence of psychiatric disorders and limit the development of chronic psychiatric and physical symptoms. Nonetheless, once disclosure occurs, its effects depend directly on the reactions of those surrounding the survivor (family, friends, and professionals). The support and understanding of close family and friends have been identified as helping factors in the disclosure and repair processes (44). On the other hand, reactions are sometimes non-empathetic, questioning the truthfulness of—and blaming—the survivor. In these circumstances, disclosure has a deleterious impact and increases the survivor's vulnerability. An exploratory study of the accounts of 19 women survivors of SSA by their brothers (40) reported that professionals had made a number of unhelpful responses, including that the behavior involved mere experimentation by the boy, was mutually initiated, was the victim's fault, or could not be abuse as it involved a brother. By contrast, survivors experienced professionals as extremely helpful when the latter believed the disclosure and acknowledged the abusiveness of the behavior. In our three case reports, disclosure permitted the appropriate adaptation of these adolescents' medical, psychiatric, social, and legal care. This multidisciplinary management contributed to improving the initial symptoms and sometimes to remission.

### Implications for Practice

First of all, it is crucial that healthcare providers recognize the seriousness and harmful effects of SSA on adolescents. Several attitudes from the professional can be helpful and supportive for the adolescent who is disclosing SSA (4, 43, 45–47):

-Talking naturally with the adolescent, clearly naming the violence, its severity, and its wrongness (“no one has the right to do that”);
-Valuing and supporting adolescents for the disclosure, insisting they must realize that it is not their fault, they are not responsible for anything; they are the victim;
-Showing consideration, care, support, and empathy;
-Informing the parents and professionals (child protection services, social workers, psychologists, medico-legal experts, etc.) who are involved in adolescents' treatment and protection;
-Reporting the situation to child welfare authorities. Mandatory reporting of child abuse and neglect varies between jurisdictions in Europe and in North America (48, 49), but whether legally mandated or otherwise this is an essential action to take in response to disclosure.

Due to the frequency of associated disorders, a psychiatric and pediatric assessment should be systematically provided to adolescents disclosing SSA. There is a consensus in the literature that good therapeutic interventions should be multidisciplinary and integrative and should consider the needs of the entire family, from the moment of disclosure to the end of therapy (4). Cases of SSA often require the involvement of a large number of health professionals, each working with a different family member (the perpetrator, the survivor, the family as a unit, etc.) (11). Study after study (5, 50–52) demonstrates it is more than likely that the child who harmed their sibling is one manifestation of problems within the family. Consideration of the entire family's needs, the proposal of multidisciplinary care, and involvement of the legal authorities as required, significantly improve the recovery of survivors as well as perpetrators (18), parents (4), and other siblings (53).

## Conclusion

The literature includes very few case reports of SSA (11, 31, 32), none of them in general psychiatric journals. Our case series highlights how harmful SSA is for adolescent survivors. They may manifest many different mental and physical symptoms. Listening to them and offering a protective multidisciplinary response can limit the lasting damage and contribute to the repair process. Protective multidisciplinary support post sibling sexual abuse disclosure must include: believing the adolescent, providing emotional support and intervention for the siblings and the family, and taking protective action (4, 29). Improving professional assessment urgently requires a better understanding of what helps these adolescents to disclose. Future studies of SSA disclosure would be beneficial for this purpose. Qualitative research can be useful on this sensitive topic (54) and can provide direct access to experiences of adolescents, parents, and healthcare professionals concerned about the disclosure of SSA.

## Data Availability Statement

The original contributions presented in the study are included in the article/supplementary material, further inquiries can be directed to the corresponding author/s.

## Ethics Statement

Ethical review and approval was not required for the study on human participants in accordance with the local legislation and institutional requirements. Written informed consent to participate in this study was provided by the participants' legal guardian/next of kin. Written informed consent was obtained from the minor(s)' legal guardian/next of kin for the publication of any potentially identifiable images or data included in this article.

## Author Contributions

EC conceived the case study and wrote the initial manuscript. NF contributed to the literature review. SG, JL, and MM edited the manuscript. All authors contributed to the article and approved the submitted version.

## Conflict of Interest

The authors declare that the research was conducted in the absence of any commercial or financial relationships that could be construed as a potential conflict of interest.

## Publisher's Note

All claims expressed in this article are solely those of the authors and do not necessarily represent those of their affiliated organizations, or those of the publisher, the editors and the reviewers. Any product that may be evaluated in this article, or claim that may be made by its manufacturer, is not guaranteed or endorsed by the publisher.
